# Calcification of intervertebral discs in the dachshund: a radiographic and histopathologic study of 20 dogs

**DOI:** 10.1186/1751-0147-49-39

**Published:** 2007-12-21

**Authors:** Øyvind Stigen, Øyvor Kolbjørnsen

**Affiliations:** 1 Department of Companion Animal Clinical Sciences, Norwegian School of Veterinary Science, PO Box 8146, 0033 Oslo, Norway; 2 Department of Pathology, National Veterinary Institute, PO Box 8156, 0033 Oslo, Norway

## Abstract

**Background:**

The purpose of the study was to compare radiographic and histopathologic findings with regard to number and extent of calcified discs in the dachshund.

**Methods:**

The intervertebral discs of 20 dachshunds were subjected to a radiographic and histopathologic examination. The dogs were selected randomly from clinical cases euthanased for reasons unrelated to research at the Norwegian School of Veterinary Science. Lateral radiographs were taken of the vertebral columns after removing them from the carcasses. The histopathologic examination included 5 μm thick sections in the transverse plane, stained with hematoxylin-eosin and von Kossa. Radiographs and histological sections were evaluated independently.

**Results:**

A total of 148 (28.5%) calcified discs were identified at the radiographic and 230 (45.7%) at the histopathologic examination. Of 92 discs found to be calcified by histopathology, but not by radiography, the degree of calcification was evaluated as 'slight' in 84 (91.3%). All the intervertebral discs (n = 138) that were found to be calcified by radiography were also found to be calcified by histopathology.

**Conclusion:**

A sensitivity of 0.6 and specificity of 1.0 for radiography was calculated when using histopathology as the gold standard.

## Background

Intervertebral disc disease (IDD) is the most common cause of neurologic dysfunction in the dog [1]. The disease is most often diagnosed in dachshunds, and in 8117 canine cases of IDD, Priester [2] found 3898 (48.0%) to occur in this breed. The breed prevalence of IDD in the dachshund has been estimated to be 19.0% [3].

In dachshunds and other chondrodystrophoid breeds, IDD is mainly due to extrusion of nuclear material from the disc into the vertebral canal. Hansen [4] classified this type of disc disease as protrusion of type 1 and found chondroid degenerative changes of the disc with subsequent dystrophic calcification of the nucleus to be significant preceding factors. In accordance with this, radiographic studies have shown that young dachshunds with calcified intervertebral discs are predisposed to IDD [5,6].

The occurrence of calcified discs is found to differ between dachshunds of the same age, size and coat variety [7]. Furthermore, a genetic factor is shown to be essential for the occurrence of calcified discs in a dog and the heritability in Norwegian dachshunds is estimated to be 0.15 and 0.22 [8]. In Danish wirehaired dachshunds Jensen and Christensen [9] estimated the heritability to be 0.60 and 0.87. Thus, the potential exists for a breeding programme that could reduce the occurrence of calcified discs and thereby IDD in this breed.

Low-field magnetic resonance (MR) imaging is found to be very sensitive in the evaluation of disc degeneration in dogs [10]. However, the cost of MR imaging is high and the number of MR machines for veterinary usage is limited. For screening purposes, a radiographic examination is therefore still the most suitable method for the identification of calcified discs in a large number of live dachshunds. Information obtained by reading radiographs can be included as part of a breeding programme.

Different degrees of calcification are found in degenerated chondrodystrophoid discs [11,12]. Discs with minor calcifications could be difficult to identify on radiographs. Also, non-calcified discs could be misjudged and read as calcified. By this, false-positive and false-negative errors may be made in reading radiographs for calcified discs. To the authors' knowledge the extent of such errors has not been reported. However, in a pathologic study of 16 one-year old chondrodystrophoid dogs Hansen [4] found calcified discs in at least 10 (62.5%) of the dogs, while in a radiographic study of 327 one-year old dachshunds Stigen [7] found calcified discs in 79 (24.2%) of the dogs. On the assumption that most of the dogs in the former study were dachshunds, these two studies together indicate that a radiographic study is far less sensitive than a pathologic study in identification of calcified discs. The present study was performed to test this hypothesis.

## Methods

The vertebral columns were obtained from 20 dachshunds that were euthanased for reasons unrelated to research at the Department of Companion Animal Clinical Sciences, Norwegian School of Veterinary Science. The case material, including size and coat varieties, sex, age and reason for euthanasia is presented in Table 1. The age of the dachshunds ranged from ten months to 13 years (mean 5.3 years) and there were twelve (60.0%) females.

**Table 1:** Case material.

Dog no.	Variety	Sex	Age, years	Reason for euthanasia
				
	Size, Coat			
1	Dwarf, Smoothcoated	♀	8	Neurological disorder
2	Dwarf, Longhaired	♀	<1 (10 months)	Vascular ring anomaly
3	Dwarf, Longhaired	♂	5	Intoxication
4	Dwarf, Longhaired	♀	6	Neurological disorder
5	Dwarf, Longhaired	♂	6	Multiple fractures
6	Dwarf, Longhaired	♀	7	Multiple fractures
7	Dwarf, Longhaired	♀	13	Pelvic fracture
8	Standard, Smoothcoated	♂	<1 (10 months)	Neurological disorder
9	Standard, Smoothcoated	♀	5	Neurological disorder
10	Standard, Smoothcoated	♂	6	Neurological disorder
11	Standard, Smoothcoated	♂	8	Complications to perineal hernia
12	Standard, Wirehaired	♂	<1 (11 months)	Owner's request
13	Standard, Wirehaired	♀	2	Owner's request
14	Standard, Wirehaired	♀	4	Neurological disorder
15	Standard, Wirehaired	♀	4	Neurological disorder
16	Standard, Wirehaired	♀	5	Pulmonary neoplasm
17	Standard, Wirehaired	♀	7	Neurological disorder
18	Standard, Wirehaired	♂	10	Owner's request
19	Standard, Longhaired	♂	3	Multiple fractures
20	Standard, Longhaired	♀	4	Neurological disorder

The vertebral columns were separated from the skull, ribs and pelvis by dearticulation. The tail, including the coccygeal vertebrae, was also removed. The spines were freed of muscle, loose connective tissue and ligaments.

Within 24 hours of death, conventional lateral radiographs were taken of the cervical, thoracic, lumbar and sacral vertebral columns [13]. The radiographic equipment was a Philips Medio 50 CP generator and a Super Rotalix 2550 tube. The cassettes were Cawo 18 × 24 cm rectangle x-ray cassettes containing a green-emitting 100 MR (fine) intensifying screen on one side of a Kodak T-Mat L/RA-film. No grid was used and at exposure the specimens were in direct contact with the cassettes. The voltage and milliampere-seconds used depended upon the size of the vertebrae and varied between 50 and 60 kV and 10 and 20 mAs respectively.

**Figure 1:**
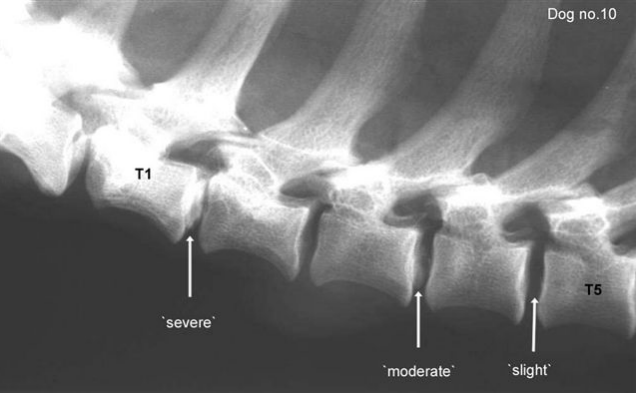
Lateral radiograph from the seventh cervical (C7) to the fifth thoracic (T5) vertebra in a six-year-old, male, smoothcoated dachshund of standard size (Dog no. 10). Three intervertebral discs; T1–2, T3–4 and T4–5, shows a 'severe', 'moderate' or 'slight' degree of calcification (arrows).

At least four exposures were taken of each dog covering the vertebral column from the first cervical (C1) to the first sacral (S1) vertebra. The total number of calcified discs and their location in the vertebral column were recorded according to earlier described methods [13,14]. Three degrees of calcification of the individual discs were noted and defined as follows (Figure 1):

SLIGHT:

- one or more calcified bodies with diameter less than 2 mm, or

- calcification of part of the transitional zone between the nucleus pulposus and the annulus fibrosus, or

- indistinct calcification of a larger part of the nucleus pulposus

MODERATE:

- calcified bodies with diameter less than 2 mm *and *calcification of part of the transitional zone between the nucleus pulposus and the annulus fibrosus, or

- distinct calcification of a larger part of the nucleus pulposus, or

- indistinct calcification of the entire nucleus pulposus

SEVERE:

- distinct calcification of the entire nucleus pulposus

If there was doubt about calcification or the degree of calcification, additional radiographs were taken of the vertebral section of current interest.

Immediately after the radiographic examination, specimens for histological examination were collected by removing each intervertebral disc between the second cervical (C2) and first sacral (S1) vertebra in all of the 20 vertebral columns. With tissue forceps, a scalpel handle and no. 11 blade the discs were carefully separated from the adjacent vertebral body end plates (*extremitas cranialis et caudalis*) and removed.

All specimens (520 discs) were fixed in 4% phosphate-buffered formaldehyde, pH 7.2. Subsequently the discs were dehydrated in ethanol, equilibrated in xylene and embedded in paraffin. Decalcifications of the specimens were not performed. The discs were sectioned transversely at about 5 μm and stained with hematoxilin-eosin and von Kossa, a method for demonstration of calcium [15]. During the preparation process all sections concerning 17 discs became incomplete and consequently these discs were regarded as withdrawals. By this, 503 discs were available for a final histopathologic examination by light microscopy.

Three degrees of calcification of the individual discs were noted, and for each category, all findings might appear either separately or in combination with other lesions. The degrees of calcification were defined as follows:

SLIGHT (Figure 2):

**Figure 2:**
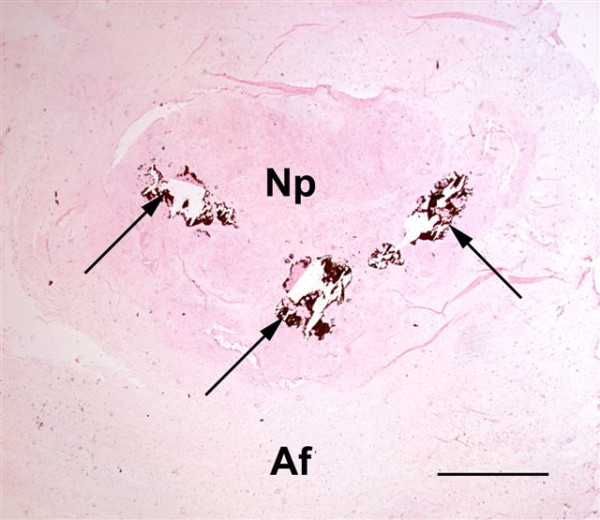
Histopathology of disc no. 6 (C7–T1) from Dog no. 10. Calcium deposits (arrows) are seen multifocally occupying less than half of the area of the nucleus pulposus (Np): 'slight' degree of calcification (**1**). Af = annulus fibrosus. Von Kossa; Bar = 1.6 mm.

- single or multiple foci of calcification occupying less than half of the area of the nucleus pulposus

- single or multiple small foci of calcification within the annulus fibrosus

**Figure 3:**
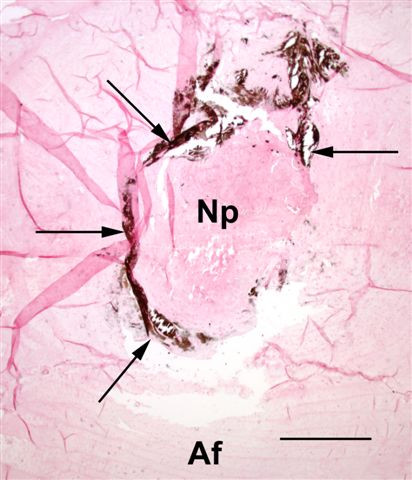
Histopathology of disc no 10 (T4–5) from Dog no. 10. Calcium deposits (arrows) form a thin discontinuous ring surrounding the nucleus pulposus (Np), with one focus extending into the annulus fibrosus: 'moderate' degree of calcification (**2**). Af = annulus fibrosus. Von Kossa; Bar = 1.6 mm.

MODERATE (Figure 3):

- single or multiple foci of calcification occupying more than half, but not all, of the area of the nucleus pulposus

- single or multiple medium size foci of calcification within the annulus fibrosus

- thin calcified ring, continuous or discontinuous, either surrounding the nucleus pulposus or localised within the annulus fibrosus

SEVERE (Figure 4):

**Figure 4:**
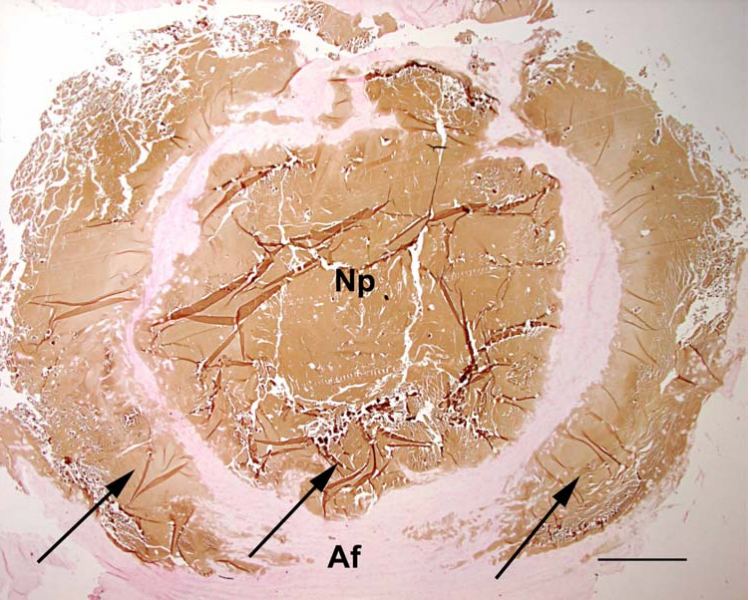
Histopathology of disc no. 7 (T1–2) from Dog no. 10. Calcium deposits (arrows) in the nucleus pulposus (Np) and in a broad discontinuous ring within the annulus fibrosus (Af): 'severe' degree of calcification (**3**). Von Kossa; Bar = 1.6 mm.

- widespread or total calcification of the nucleus pulposus

- single or multiple large foci of calcification within the annulus fibrosus

- broad calcified ring, continuous or discontinuous, either surrounding the nucleus pulposus or localised within the annulus fibrosus

If two or more sections of the same disc were read to have different degrees of calcification, the section with the most severe degree of calcification determined the evaluation of that disc.

Radiographs and histological sections were evaluated independently.

## Results

At the radiographic examination, calcification was found in 148 (28.5%) of the 520 discs. Of all affected discs, a severe degree of calcification was found in 15, a moderate degree in 43 and a slight degree in 90 discs. Calcified discs were identified in 18 (90.0%) of the dogs and the number of calcified discs in each affected dog ranged from one (dog no. 19) to 20 (dog no. 15).

At the histopathologic examination, calcification was found in 230 (45.7%) of the 503 discs. Of all affected discs, a severe degree of calcification was found in 30, a moderate degree in 45 and a slight degree in 155 discs. Calcified discs were identified in all the dogs and the number of calcified discs in each dog ranged from one (dog no. 19) to 24 (dog no. 17).

The individual number and location of calcified discs together with the degree of calcification of each affected disc, identified both by radiography and histopathology, are presented in Additional file 1, Table 3.

Comparative results by radiographic and histopathologic examination concerning 503 discs are presented in Table 2. Of all the discs found to be calcified by histopathology (n = 230), 138 (60.0%) were found to be calcified by radiography. All the discs found to be calcified by radiography (n = 138) were also found to be calcified by histopathology. Using histopathology as the gold standard, a sensitivity of 0.6 (138/230) and a specificity of 1.0 (273/273) was calculated for the radiographic examination.

**Table 2:** The distribution of 503 intervertebral discs on the basis of calcification (+) or not (-) evaluated by a radiographic and a histopathologic examination.

		Histolopathology		Total
			
		+	-	
Radiology	+	138 (27.4%)	0	138 (27.4%)
	-	92 (18.3%)	273 (54.3%)	365 (72.6%)

Total		230 (45.7%)	273 (54.3%)	503 (100.0%)

In 95 discs the degree of calcification was found to be equal in histopathology and radiography. In 125 discs the degree of calcification was higher in histopathology and in 10 it was higher in radiography.

## Discussion

The present study shows that a significantly higher number (45.7% versus 27.4%) of calcified discs are identified by a histopathologic examination compared to a radiographic examination of intervertebral discs in dachshunds. This finding verifies the hypothesis presented at the beginning of the present paper.

At the radiographic examination, a total of 148 calcified discs were identified in 18 dogs. This makes a mean of 8.2 calcified discs in each affected individual. Both the percentage number of dogs with calcified discs (90.0%) and the mean number of such discs in each affected dog are higher than values for corresponding parameters found in previous radiographic studies [6,7,12]. The fact that the present study is related to clinical cases, whereas the previous radiographic studies were related to clinically normal dachshunds, could be the reason for this disparity.

In dogs no. 2 and 3, no calcified discs were found at the radiographic examination, but at the histopathologic examination a slight degree of calcification was found in five and 15 discs, respectively. This finding shows that a total absence of calcified discs visible on radiographs of a dachshund is no guarantee for the dog not having extensive calcification at histopathologic examination.

Of the 92 discs that were found to be calcified only at the histopathologic examination, 84 (91.3%) were noted to have a 'slight' degree, eight a 'moderate' degree and none a 'severe' degree of calcification. This agrees with the general understanding that an ordinary radiographic examination of the vertebral column in dogs is not very sensitive at detecting minor calcifications of intervertebral discs.

The radiographic examination in the present study was done on vertebral columns separated from the skull, ribs and pelvis and freed of soft tissue. This meant that superimposition of tissue adjacent to the discs (e.g. *caput costae *and *os ilium*) was avoided on the radiographs. Also motion blurring was not a problem with the examination of dead specimens. These factors contributed to high technical quality of the radiographs. Therefore, the sensitivity of the radiographic examination in the present study is likely higher than could be expected in similar studies on live dogs. As a consequence, the presented results concerning total number of calcified discs and degree of calcification of individual discs, cannot directly be compared to results from previous radiographic studies [5-7,9,12] on live dogs.

The 20 dachshunds included in the present study were euthanased up to 24 hours before being available for the radiographic examination. As a result, *rigor mortis *was present in several dogs and for these the standard procedure for spinal radiography of live dogs [13,16] was inappropriate. Two dogs (no. 3 and 18) had been subjected to post-mortem examinations immediately after euthanasia and their vertebral columns were thereafter included in the study. To achieve radiographs of high technical quality from more dogs, separated vertebral columns, instead of vertebral columns "in situ", were chosen for radiography. Diagnostic imaging of separated vertebral columns is previously reported in radiographic [17,18] and MR [10] examinations in dogs.

Radiographs and histological sections were each evaluated by one person, which means that both the radiographic and the histopathologic examination were subject to significant interobserver variation. With two or more persons evaluating the radiographs and the histological sections respectively, the interobserver variation and consequently the standard deviations of the observations presented in Table 2, would decrease. Consequently, the calculated sensitivity of 0.6 represents an underestimate.

At the radiographic examination, additional radiographs were taken if there was any doubt about calcification. At the histopathologic examination, at least two complete histological sections of each disc were examined to remove any doubt about calcification. By this, the present study should not be encumbered with significant intraobserver variation.

A specificity of 1.0 indicates that no false-positive errors were made at the radiographic examination. The high technical quality of the radiographs is probably a significant reason for this. Another reason could be a reserved attitude by the reader of the radiographs, evaluating discs with uncertain calcification as negative.

Von Kossa, a special stain for identification of calcium, was included in the histopathologic examination of all intervertebral discs. In previous histopathologic examinations of disc degeneration in dogs [4,10], this stain was not used. In the study by Seiler *et al*. [10], decalcification was also part of the preparation for histopathologic examination. A discrepancy in results can easily arise when different studies adopt different protocols.

In the present study, histopathology was found considerably more sensitive than radiography in identifying calcified discs. Nevertheless, in ten discs the degree of calcification was found to be higher in radiography than in histopathology. A histopathologic examination of non representative sections could be an explanation for the different findings in these ten discs.

## Conclusion

A histopathologic examination is superior to a radiographic examination in identification of calcified intervertebral discs in the dachshund. A sensitivity of 0.6 and a specificity of 1.0 for radiography is calculated using number of calcified discs as parameter and histopathology as the gold standard. Calcification of the nucleus pulposus and/or the annulus fibrosus is a significant sign of disc degeneration in dachshunds and the distribution of detectable calcification in affected discs represents the distribution of a continuous variable.

## Competing interests

The author(s) declare that they have no competing interests.

## Authors' contributions

ØS conceived of the study, carried out the radiographic examination and is the main author of the paper. ØK carried out the histopathologic examination. Both authors read and approved the final manuscript.

## Supplementary Material

Additional file 1 Table 3. Degree of calcification, evaluated by a radiographic and a histopathologic examination in 26 intervertebral discs from each of 20 dachshunds. Degrees of calcification evaluated by radiography: light grey: slight. dark grey: moderate. black: severe. Degrees of calcification evaluated by histopathology: **1 **slight. **2 **moderate. **3 **severe. Other note: **-: **intervertebral disc not available for a final histopathologic examination. Click here for file
