# Supplementary material for: Calcification of intervertebral discs in the dachshund: a radiographic and histopathologic study of 20 dogs
Source: Acta Vet Scand. 2007 Dec 21;49(1):39. doi: 10.1186/1751-0147-49-39 (PMC2262089; doi:10.1186/1751-0147-49-39)
Supplement: Additional file 1 — Table 3. Degree of calcification, evaluated by a radiographic and a histopathologic examination in 26 intervertebral discs from each of 20 dachshunds. Degrees of calcification evaluated by radiography: light grey: slight. dark grey: moderate. black: severe. Degrees of calcification evaluated by histopathology: 1 slight. 2 moderate. 3 severe. Other note: -: intervertebral disc not available for a final histopathologic examination. [file 1751-0147-49-39-S1.doc]

| Disc no. Dog no. | 1 | 2 | 3 | 4 | 5 | 6 | 7 | 8 | 9 | 10 | 11 | 12 | 13 | 14 | 15 | 16 | 17 | 18 | 19 | 20 | 21 | 22 | 23 | 24 | 25 | 26 |
| --- | --- | --- | --- | --- | --- | --- | --- | --- | --- | --- | --- | --- | --- | --- | --- | --- | --- | --- | --- | --- | --- | --- | --- | --- | --- | --- |
| 1 |  |  |  |  |  | **1** |  |  |  |  |  |  |  | **2** |  |  | **2** |  | **2** | **-** |  |  |  |  |  |  |
| 2 |  |  |  |  |  |  | **1** |  | **1** | **1** |  | **1** | **1** |  |  |  |  |  |  |  |  |  |  |  |  |  |
| 3 |  | **1** |  | **1** | **1** | **1** | **1** | **1** | **1** | **1** |  | **1** | **1** | **1** |  |  |  | **1** |  | **1** |  | **1** | **1** |  |  |  |
| 4 |  | **1** | **1** |  |  | **1** | **1** | **-** |  | **-** | **2** | **-** | **3** | **-** | **1** | **-** | **1** | **3** | **3** | **-** | **-** |  |  | **-** | **3** | **2** |
| 5 |  | **1** |  |  |  | **3** |  | **1** | **-** |  | **1** | **2** | **1** | **1** | **1** | **2** |  | **1** |  | **1** | **1** |  |  |  |  |  |
| 6 | **1** |  |  |  | **1** | **1** | **2** | **1** | **3** | **1** | **1** |  | **1** | **2** | **2** | **1** | **1** |  | **1** | **1** |  |  |  | **1** | **2** |  |
| 7 |  |  |  |  |  |  |  |  |  |  |  |  |  |  |  |  |  | **1** | **1** | **1** |  |  | **2** |  | **1** |  |
| 8 |  | **1** |  | **1** |  | **1** | **2** | **1** |  |  |  | **1** | **2** | **2** | **1** | **2** |  | **2** |  |  |  |  |  |  |  |  |
| 9 |  | **1** | **1** |  |  | **1** |  |  |  |  |  |  | **1** |  |  | **3** | **3** |  |  |  |  |  |  |  |  |  |
| 10 |  |  |  |  |  | **1** | **3** | **1** | **3** | **2** | **2** |  | **1** | **1** | **1** | **1** | **2** | **3** | **1** |  | **3** |  |  |  |  |  |
| 11 |  |  |  |  |  | **1** |  | **1** |  |  |  |  | **1** | **-** | **-** |  |  | **-** |  | **1** | **-** |  |  |  |  |  |
| 12 |  | **1** |  |  |  |  | **1** |  | **3** |  | **1** | **1** | **1** | **3** | **1** | **1** | **3** |  | **1** | **1** | **1** |  |  | **1** |  | **2** |
| 13 |  |  |  |  |  |  | **3** | **1** | **3** | **2** | **1** |  | **1** | **-** | **2** | **3** | **1** |  |  |  |  |  |  |  |  |  |
| 14 | **1** |  | **1** | **2** | **1** | **3** | **1** | **1** | **1** | **3** | **1** | **1** | **1** | **1** | **1** | **3** | **1** |  |  |  | **1** |  | **2** | **1** | **1** | **2** |
| 15 | **1** | **1** | **1** | **2** |  | **1** | **2** | **1** | **1** | **1** | **2** | **3** | **3** | **3** | **1** | **1** |  | **1** | **2** | **1** | **1** | **1** | **1** | **1** | **2** | **3** |
| 16 |  |  |  |  |  |  |  |  | **2** | **2** | **1** | **2** | **1** | **1** |  |  |  |  |  |  |  |  |  |  |  |  |
| 17 | **2** | **1** | **1** | **3** | **1** | **1** | **1** | **1** | **1** | **2** | **1** | **1** | **2** | **1** | **2** | **1** | **3** | **3** |  | **1** |  | **2** | **1** | **1** | **1** | **2** |
| 18 |  |  | **2** | **1** |  | **2** |  |  |  | **-** |  |  |  |  | **2** |  |  |  |  |  | **1** |  |  |  |  |  |
| 19 |  |  |  |  |  | **1** |  |  | **-** |  |  |  |  |  |  |  |  |  |  |  |  |  |  |  |  |  |
| 20 | **1** | **1** | **1** |  | **1** |  | **3** | **1** |  | **2** | **1** | **1** |  | **1** | **2** | **1** | **1** | **1** | **3** | **1** | **1** | **1** |  |  | **1** | **1** |
